# The Heart of the Matter: Staff Insights Into Organ Donation Conversations

**DOI:** 10.1111/nicc.70668

**Published:** 2026-09-08

**Authors:** Lydia Shim, Cynthia J. Wensley, Rachael L. Parke

**Affiliations:** ^1^ Department of Critical Care Medicine Te Toka Tumai Auckland, Grafton Auckland New Zealand; ^2^ School of Nursing The University of Auckland, Faculty of Medical and Health Sciences, Grafton Auckland New Zealand; ^3^ Cardiothoracic and Vascular Intensive Care Unit Te Toka Tumai Auckland, Grafton Auckland New Zealand

**Keywords:** donation conversations, focus groups, healthcare professionals, intensive care units, organ donation

## Abstract

**Background:**

Organ donation in intensive care units (ICU) involves complex, emotional conversations with families during end‐of‐life care. While literature often examines consent rates, clinical protocols and family experiences, less is known about the factors that shape donation discussions from the perspective of ICU staff.

**Aims:**

To understand ICU staff experiences and perspectives of factors influencing organ donation discussions and family decision‐making in New Zealand ICUs.

**Study Design:**

A qualitative descriptive study using reflexive thematic analysis of focus group data from 11 ICU staff (medical, nursing and health psychology) across three tertiary ICU facilities.

**Findings:**

Three key themes were developed: *Having a ‘Good’ Conversation*, *Acknowledging the Emotional and Mental Weight and Having the Will to Endure* and *Feeling Equipped to Care*. Participants emphasised the importance of trust‐based communication with families; culturally and emotionally responsive care tailored to families' individual needs; and regulating the emotional demands of donation work. Staff relied on regular collegial support to maintain emotional resilience and valued structured education, team preparation and access to resources that built their confidence in donation conversations. Participants identified practices and values that fostered a positive unit culture around organ donation, contributing to team resilience and meaningful family interactions.

**Conclusions:**

This study offers insight into the interpersonal, emotional and practical aspects of organ donation work in NZ ICUs from the perspective of staff. Findings show that supportive team culture, underpinned by quality communication, team preparation, emotional safety and adequate resources, enables more meaningful donation conversations with families. These insights highlight opportunities for strengthening practice, education and unit culture in donation work.

**Relevance to Clinical Practice:**

Findings support the need for regular, multidisciplinary education; emotional support structures; and clear, standardised team processes to enhance donation conversations. Improving systemic infrastructure, such as robust donor identification pathways and public‐facing education, can further prepare staff and families to navigate donation as a normalised aspect of end‐of‐life care.

## Impact Statements

- What is known about the topic
  ○ Organ donation conversations in ICUs are emotionally and ethically complex.
  ○ Staff often experience stress and uncertainty when supporting families through donation decisions.
- What this paper adds
  ○ Emphasises the importance of early, inclusive communication that centres around family trust over consent outcomes.
  ○ Acknowledges the emotional weight experienced by staff involved in organ donation care and recognises the importance of regulating emotional loads through normalising emotional expression and formal and informal collegial support to safeguard staff well‐being and endurance.
  ○ Offers practical and systemic insights to support well‐informed organ donation conversations and decision‐making and a positive donation culture in ICU settings.

## 1 Introduction

Intensive care unit (ICU) staff play a crucial role in organ donation conversations with families [1], often in high‐stress situations involving complex emotional, ethical and cultural factors that continue to evolve until donor surgery [2, 3]. Challenges including emotional burden, unclear protocols and communication difficulties highlight the need for greater support in providing sensitive, effective assistance to families [4, 5]. Understanding ICU staff experiences is essential to optimising communication with families, supporting staff well‐being and improving the donation experience [6].

## 2 Background

The clinical priorities of organ donation involve identifying potential donors, assessing eligibility and maintaining organ viability [4]. Within limited timeframes, ICU staff must also support families through accepting death while facilitating sensitive donation conversations [5]. However, staff confidence, varying institutional protocols and navigating family dynamics can pose challenges [5, 7]. An integrative literature review [8] highlights four domains influencing ICU staff in this context: knowledge and training, communication, internal factors (personal values and emotions) and external factors (environment and organisational resources).

Cultural considerations are important in multicultural healthcare settings such as New Zealand (NZ), which promotes culturally safe, equitable care [9, 10]. Guided by Te Tiriti o Waitangi (The Treaty of Waitangi), NZ healthcare incorporates Indigenous Māori values, including collective decision‐making and holistic views of death and autonomy, influencing donation conversations [10, 11].

In NZ, organ donation follows an opt‐in system [12] coordinated nationally by Organ Donation New Zealand (ODNZ) [13]. Specialist donor coordinators support donor assessment, communication between ICU teams and transplant services and families throughout the donation process [13]. NZ undertakes both donation after neurological determination of death (DNDD) and donation after circulatory determination of death (DCDD), with DCDD representing a growing proportion of donations [13]. In 2024, 44% of families approached consented to donation [13].

NZ guidelines recommend donation discussions occur after death or decisions to withdraw life‐sustaining treatment [12]. ICU clinicians typically lead these discussions [12, 14], with ODNZ providing specialist support to staff and families [15]. Donation conversations are clinically and culturally complex, requiring ICU staff to navigate diverse worldviews, family dynamics and the demands of potential donor and end‐of‐life care [5, 7, 16, 17]. While extensive research has examined healthcare professionals' experiences, few have exclusively explored ICU staff perspectives on factors shaping bedside donation discussions [18]. Understanding these experiences may inform best practice and strengthen family‐centred organ donation decision‐making.

## 3 Research Aim

To understand ICU staff experiences and perspectives of factors influencing organ donation discussions and family decision‐making in NZ ICUs.

## 4 Design and Methods

### 4.1 Study Design

This qualitative descriptive study used focus groups and reflexive thematic analysis (RTA) [19], following the Reflexive Thematic Analysis Reporting Guidelines (RTARG) [20]. The theoretical flexibility of RTA supports deductive and inductive analysis [19] and aligns with the study's critical realist [21] and pragmatist [22] paradigms. The primary researcher (L.S.), an ICU nurse, used clinical experience to build rapport, guide discussion and manage group dynamics.

### 4.2 Setting

The study was conducted between November 2024 and March 2025 across three metropolitan, tertiary‐level ICUs within two hospitals in NZ.

### 4.3 Participant Recruitment and Characteristics

Purposive sampling [23] was used. ICU managers emailed study invitations to doctors, nurses and health psychologists in participating ICUs. Flyers with QR codes linking to study details were displayed at ICU sites. Interested participants contacted the research team directly. Table 1 presents the eligibility criteria.

**TABLE 1:** Eligibility criteria for participants.

Discipline	Eligibility criteria
Applicable to all disciplines	Have experienced at least one of the following in a NZ ICU: Initiating a discussion on donation after death with family/whānau Participating in a discussion on donation after death with family/whānau Supporting family/whānau through a decision on donation after death
Doctors	Currently practising at registrar level or above in the ICU
Nurses	Registered nurse with > 10 weeks ICU experience (i.e., completed ICU orientation and commenced solo shifts)
Health psychologists	Eligible if they have met at least one of the shared experience criteria listed above.

*Note:* Health psychologists do not routinely participate in organ donation discussions but may become involved at the request of families/whānau or ICU staff.

Eleven participants (four doctors, five nurses, two health psychologists) participated in four focus groups and one interview (after a late cancellation) to discuss their experiences with both DNDD and DCDD. Table 2 presents participant characteristics.

**TABLE 2:** Participant characteristics (*n* = 11).

Characteristic	Number of participants (*n*)
Profession	
Doctors	4
Nurses	5
Health psychologists	2
Gender	
Female	8
Male	3
Ethnicity (self‐described)	
NZ European	9
Sri Lankan (South Asian)	1
Filipino (Southeast Asian)	1
Age (years)	
30–39	6
40–49	4
50–59	1
ICU experience (years)	
< 1	1
1–5	5
6–10	1
> 10	4
Number of donation conversations	
2–5	4
6–10	1
> 10	6
Donation‐specific training received (participants could select more than one option)	
NZ Donation Awareness Course	5
Core Donation Family Workshop (cFDC)	6
Organ Donation Study Day	9
ICU Donation Audit presentations	4
Advanced study day	5
Induction study day for Link nurses	2
None	1

*Note:* Organ Donation New Zealand provides donation education and training for health professionals across New Zealand [13]. Courses and workshops include education on the donation process, family donation conversations, donor identification and professional roles within donation care. Study days provide broader awareness of organ and tissue donation, transplantation and current donation practice [13].

### 4.4 Data Generation

Data were collected by L.S. using a semi‐structured topic guide (File S1) informed by an integrative review [8]. Minor revisions were made after piloting with two ICU nurses to improve clarity and relevance.

Post‐focus group journaling and observational notes documented group dynamics and non‐verbal cues to support reflexivity and consideration of researcher influence. Data collection ceased based on information power [24], as participants' insights became sufficiently rich and aligned to the study aim.

Inclusive facilitation techniques encouraged diverse perspectives, and potential power imbalances were minimised by conducting focus groups separately by profession. Sessions (via Zoom) lasted 67–90 min (average 76 min), were audio‐recorded and professionally transcribed.

### 4.5 Data Analysis

Data familiarisation involved repeated transcript reading alongside journal notes, supported by NVivo 15. Initial coding followed RTA [19], combining deductive coding (using a framework derived from the integrative review [8]) and inductive coding to capture novel concepts. New, inductive codes were prompted through repeated patterns of meaning, or salient participant insights not captured by the existing framework.

Both semantic and latent coding approaches were used. Semantic coding [19] captured explicit content using participants' phrases such as ‘valuing relationships’ and ‘supporting inclusion’ as code names. Latent coding [19] interpreted underlying emotional and moral meanings by drawing on tone, pauses, hesitations, facial expressions and other non‐verbal cues noted by the researcher.

Initial coding revealed participants' strengths‐focused outlook and commitment to fostering a positive organ donation culture. This informed a strengths‐based approach to theme development to align with participants' attitudes, highlighting positive experiences, resilience factors and practices supporting quality donation discussions.

### 4.6 Ensuring Trustworthiness

Credibility was established through purposive, voluntary sampling of participants with relevant experience and transparent documentation of RTA phases. Reflexivity was maintained through journaling and regular debriefings with co‐researchers (C.W. and R.P.), enabling L.S. to reflect on researcher's positionality, biases, assumptions and interpretations. Collaborative analysis enhanced rigour and ensured themes reflected participant experiences rather than researcher assumptions.

### 4.7 Ethical Approval

Ethical approval was granted by the Auckland Health Research Ethics Committee, Auckland, [Ref: AH26759, Approval Date: 18/12/2023], with locality approval obtained from participating hospitals. All participants received written information about the study and were given the opportunity to ask questions prior to participation.

## 5 Findings

**FIGURE 1:**
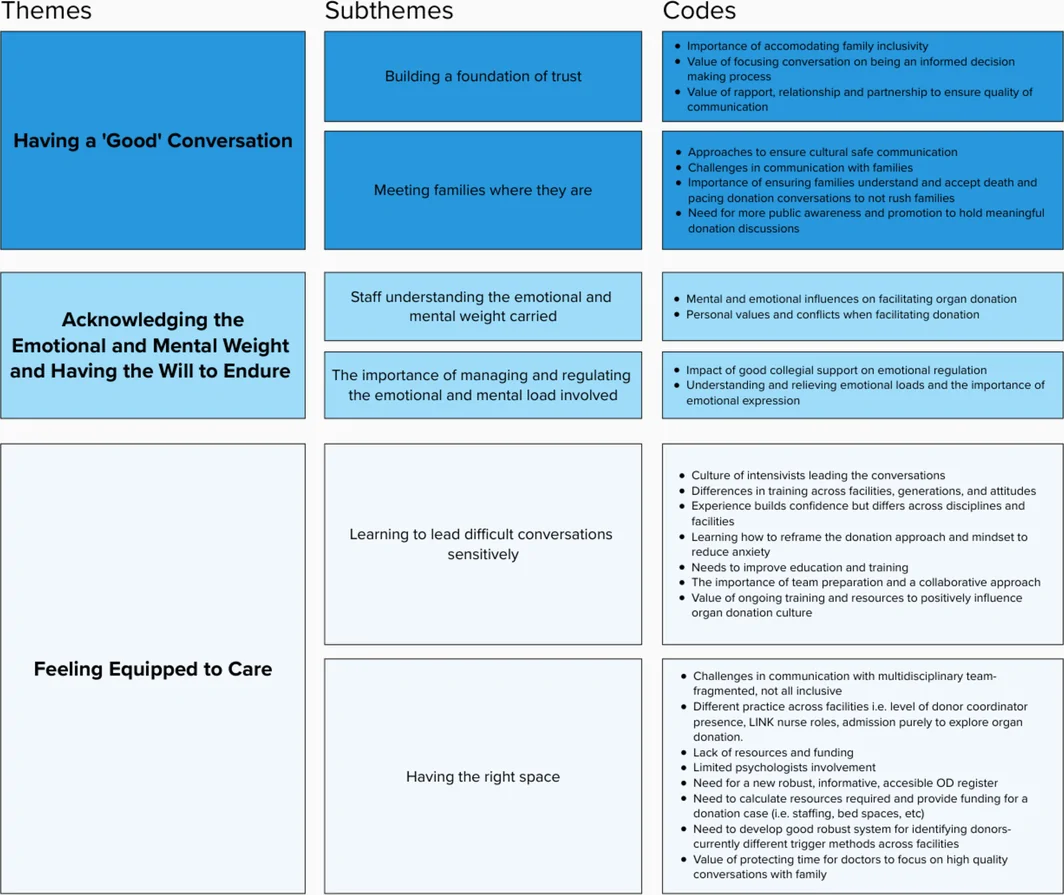
Summary of reflexive thematic analysis.

Three themes comprising six subthemes were developed: *Having a ‘Good’ Conversation*, *Acknowledging the Emotional and Mental Weight and Having the Will to Endure* and *Feeling Equipped to Care* (Figure 1). These themes frame a positive unit culture for organ donation discussions that staff identified as foundational to high‐quality practice. This culture supports emotionally resilient teams and meaningful, family‐centred discussions that extend beyond consent, prioritising communication, rapport and culturally responsive care. The themes highlight the clinical, personal, interpersonal and systemic factors that sustain this complex work.

### 5.1 Having a ‘Good’ Conversation

This theme comprised two subthemes: *Building a Foundation of Trust* and *Meeting Families Where They Are*. They describe the relational and responsive approaches staff viewed as essential to meaningful organ donation discussions. A ‘good’ conversation was grounded in trust, empathy and family‐centred communication. Staff emphasised the need to meet families where they were culturally, clinically and emotionally. Success was not defined by consent, but by interactions that enabled families to feel informed, supported and empowered to make enduring decisions aligned with their needs and values.

#### 5.1.1 Building a Foundation of Trust

Establishing trust with families early in the ICU admission was considered vital. Early family involvement supported their emotional well‐being and helped staff understand family values, dynamics, communication preferences and approaches to death. This foundation was essential to sensitive, effective communication throughout emotionally charged situations.

The donation conversations that have been the most straightforward […] are the ones where I've established a relationship and trust with the families beforehand […] that's such a crucial part […].(P2‐Doctor)

Trust was built through consistent, respectful engagement, beginning with practical actions such as scheduling regular family meetings and creating private, inclusive spaces. These practices signalled that families' voices mattered, fostering a sense of safety to express needs, clarify decision‐making roles and enabling staff to provide tailored support.

It's about how important are those people in that decision‐making journey, you have to let the family tell you that […]. (P3‐Doctor)

Establishing rapport with families eased staff anxiety and encouraged open dialogue. Staff used active listening, recognised emotional cues and strived to normalise intense emotions associated with sudden loss and grief. They prioritised allowing families to set the pace for donation discussions.

The phrase, ‘we'll be guided by you’ […] really sets the stage […] there's no hard and fast rules about when family meetings should happen and how they should go […] it gains trust in us as healthcare workers, […] we're going to put the family's wishes first. (P8‐Nurse)

When rapport was strong, staff felt a united sense of purpose with families and could centre care decisions on the patient's wishes.

There were lots of opinions […] we just brought the conversation back to, ‘I think what's really important in this situation is we focus on the patient, what do you think they would be thinking in this situation?’ then everyone was able to refocus […]. (P9‐Nurse)

Ultimately, staff found meaning in empowering families to make informed, thoughtful decisions that aligned with the patient's values, regardless of the outcome.

I firmly believe that it goes well when the families have had the opportunity for donation presented to them in a thoughtful and respectful way […] with the information that they need […] It's not just when it's a yes. It's that people will consider the question and put the [patient] at the centre of that decision. (P1‐Doctor)

Conversely, limited dialogue prevented families from fully exploring the donation option. Staff found this disappointing and viewed it as a missed opportunity for informed decision‐making. One participant described how dominant voices in the room prematurely ended a conversation.

the daughters and sisters were saying ‘I think she would really like to do that’ […] and then the male, he was older […] he just said no […] it wasn't one where everyone was fully informed or had the chance to consider [the patient's] views […] maybe that could have gone better. (P2‐Doctor)

#### 5.1.2 Meeting Families Where They Are

Staff valued recognising and responding to each family's emotional state, cultural background and clinical understanding with respect, adaptability and non‐judgement. Tailoring conversations helped families feel seen, safe and supported.

A key strategy involved adjusting communication styles to align with families' cultural norms. This included asking what mattered to families, involving cultural liaisons and supporting religious or spiritual practices.

It's about understanding their emotions, their culture, and what they know about the situation. Sometimes that means bringing in cultural advisors or making space for spiritual practices that are important to them. (P3‐Doctor)

Staff also reported mirroring families' demeanour to build connection and comfort:

If I have a family that's loud and jovial, I might mirror that a little bit. Similarly, you might get a reserved, quiet family, and I just kind of mirror the way they are culturally, to try and respect that. (P2‐Doctor)

These approaches from staff reflected a broader understanding of culture beyond ethnicity, encompassing values, communication preferences and individual ways of being.

Understanding each family's acceptance of death before discussing donation was also crucial. Although a general sequence was often followed (confirming and explaining death, then raising donation), staff emphasised flexibility. Staff narrated their bedside actions as they gave care, provided information in small, manageable parts and gently supported families to understand their loved one's irreversible condition without further emotional overwhelm. Recognising that families process loss differently, staff sometimes delayed donation conversations when families appeared to be struggling.

There's different sorts of relationships. It takes different people different amounts of time to process and accept […] you might see a lot of resistance from some people that are maybe finding it harder […]. (P11‐Health psychologist)

Having the same primary clinician involved throughout the process, when possible, was valued for reducing care fragmentation and promoting trust. Continuity of care helped staff recognise families' emotional readiness and personalise conversations.

I think being able to do the series of conversations yourself rather than hand over bits is really important because you can understand where the family are at and when the right time to discuss things is. (P3‐Doctor)

Conversely, staff recalled negative experiences when donation was introduced before families had processed their loved one's death. These conversations were described as ‘poorly planned’, ‘confronting’ and ‘harmful’ to both families and team relationships. One participant reflected on an approach that felt rushed and abrupt:

They said ‘look, he's had a catastrophic bleed, he's not going to live,’ – paused for maybe like three seconds‐ ‘do you reckon he wants to donate his organs?’ I wanted to die in my seat. (P6‐Nurse)

Families with pre‐existing distrust in healthcare were perceived as particularly challenging to engage, adding undue pressure on families and staff during an already difficult period. Participants recommended earlier public education to donation concepts to better prepare families and for open, informed conversations.

It is a massive decision to expect of people under pressure when they haven't done any pre‐thinking […] if there was a massive public campaign to say this is what donation looks like, this is the value it has […] that would make an impact. (P4‐Doctor)

### 5.2 Acknowledging the Emotional and Mental Weight and Having the Will to Endure

This theme explores the emotional and psychological demands of donation work and how staff sought to manage them. Two interconnected subthemes underpin this theme: *Staff Understanding the Emotional and Mental Weight Carried* and *The Importance of Managing and Regulating the Emotional and Mental Load Involved*. Building on family‐centred communication, a positive organ donation culture requires staff recognising the impact of this work and proactively creating conditions that support resilience and well‐being to sustain high‐quality donation discussions.

#### 5.2.1 Staff Understanding the Emotional and Mental Weight Carried

Staff faced psychological challenges from professional responsibility and organisational pressure to initiate donation conversations. Not approaching families required documented justification, adding further stress when family refusal was anticipated or navigating uncertainty about the right timing. Many also experienced anxiety around perceived expectations for flawless communication.

Emotional strain stemmed from witnessing intense grief, managing delays in the donation process and caring for patients and families staff felt connected to. Difficult family dynamics, experiencing transplant‐related complications or deaths and personal experiences of loss left lasting impacts and acute awareness of the hope and heartbreak associated with transplantation. These experiences led staff to approach future donation conversations with heightened emotional sensitivity. Anticipatory apprehension created ongoing challenges in balancing personal emotions with professional responsibilities.

That duality of your professional role and your personal role, sometimes they don't meet up […]. (P10‐Health psychologist)

Staff also navigated culturally and spiritually diverse views on organ donation within the workforce. Some viewed donation pragmatically, while others were influenced by personal connections to transplant recipients. Others experienced internal conflict when they shared families' religious faith in a higher power and understood families' hope for a miracle, despite recognising death as clinically inevitable.

There was a Christian family who kept praying for a miracle. I'm Christian too, so I understood their hope, but I also knew that death was inevitable. It was hard to watch, because I empathised with their need to keep praying, despite knowing what was coming. (P9‐Nurse)

Holding space for the intersection of faith and clinical fact—believing that a higher power hears all prayers, while also understanding the medical reality of irreversible brain injury and brain death—was deeply challenging. Staff had to navigate the tension between professional judgement and personal belief, particularly when they empathised with families' hope but still needed to support them through the realities of death.

The emotional and moral weight of donation conversations also extended to ethical dilemmas. Some staff questioned whether they could donate their own loved one's organs due to the prolonged emotional toll involved or expressed discomfort over the lack of control in choosing recipients. These dilemmas added complexity to their roles, requiring staff to reconcile personal perspectives with professional commitment.

Death and dying in general, […] there's diversity in terms of comfortability […] Staff are often operating from three or four different spaces of being, so they've got to manage their own uncomfortability. (P11‐Health psychologist)

#### 5.2.2 The Importance of Managing and Regulating the Emotional and Mental Load Involved

Normalising emotional expression was vital for sustaining endurance and resilience in organ donation work. Senior staff across professions encouraged openness within professional boundaries, teaching junior staff that expressing deep emotions was not only acceptable but necessary to prevent burnout and help staff accept the limits of care.

I make it very clear that if you don't feel then you're starting that process of burnout, and it's important that, if you feel something that we normalise that, and it's okay to show that you feel within the bounds of professionalism […]. (P4‐Doctor)

However, a key professional boundary remained prominent, that families should never feel responsible for consoling staff, reaffirming their professional role as supporters, not recipients, of emotional care.

Showing emotion, […] it shows them that you're human […] you've just got to always remember the number one rule, they can't console you. (P6‐Nurse)

Activities such as family time, being in nature and social connection were essential outlets for releasing emotional tension, but collegial support was viewed as the most important resource. Informal check‐ins, mentorship and shared reflection among senior and junior staff, helped process difficult cases, maintain perspective and fostered solidarity and vital encouragement to endure challenges together.

There is a sense of responsibility to one's team […] especially to junior members […]and checking in is important. (P1‐Doctor)

### 5.3 Feeling Equipped to Care

This theme conveys the knowledge, skills and resources needed to build staff confidence in supporting families and leading quality organ donation discussions. Two subthemes capture this experience: *Learning to Lead Difficult Conversations Sensitively* and *Having the Right Space*, highlighting how staff preparedness is shaped by the development of communication skills and access to supportive spaces that enable meaningful family‐centred care.

#### 5.3.1 Learning to Lead Difficult Conversations Sensitively

Leading organ donation conversations is a demanding task requiring skill and sensitivity. Targeted training and team preparation were essential for building confidence. While doctors often led these conversations due to their role and decision‐making responsibilities, participants emphasised that the most appropriately trained professional, regardless of discipline, should guide these discussions. Simulation workshops were especially valued for developing communication skills and reframing donation as supporting family choice, rather than a burdensome request, reducing staff anxiety.

Training, however, was typically mandated once, with no formal refreshers.

You could have been practising as a specialist for 30 years and have no further training in donation. (P4‐Doctor)

Participants felt this contributed to inconsistent knowledge and attitudes across professions and hospitals. For example, some doctors observed that colleagues without recent training held more negative attitudes toward organ donation, including a lack of confidence in available resources or viewing donation as additional work that was poorly supported. In contrast, those with recent education approached donation more positively.

It really requires some self‐motivation to attend another course […] I quite naively assumed that everybody thought like I did and was quite amazed at their attitudes. (P3‐Doctor)

Multidisciplinary team preparation and strong ICU‐ODNZ relationships was highly valued for effective practice and a positive unit culture toward organ donation. Familiarity with ODNZ coordinators increased staff confidence in seeking guidance, reducing uncertainty and improving coordination. Pre‐briefs involving bedside nurses and cultural or religious support, such as hospital kaumātua (Māori elders who offer cultural guidance and support), Pacific health services and chaplains, helped align teams, understand family, reduce the burden on individuals and lead donation conversations with greater sensitivity.

If our kaumātua and chaplains know the family, or if the family want them there, we can always include them in our prebriefs before talking to the family. It really helps us get on the same page, and takes some pressure off us, and makes those conversations feel more culturally safe and respectful. (P3‐Doctor)

However, such practices were inconsistently implemented across facilities, despite staff recognising their importance.

It sounds like [hospital name] has formalised things a little bit better than we have […] everyone knows that it's useful to have a pre‐brief, but whether it's done every time, I would say that we haven't got it squared away as clearly. (P4‐Doctor)

#### 5.3.2 Having the Right Space

Creating the right conditions to support organ donation discussions required more than individual preparedness. It depended on organisational structures, protected time, funding and system‐level resources. Competing ICU demands often deprioritised donation, especially without dedicated time or on‐call clinician hours allocated to facilitate donation activities.

We are a resource‐constrained speciality […] when people are under pressure, (donation is) the first thing that seems discretionary […] if you had a system where there was a certain calculated resource that went with every donation, then that would make things easier. (P4‐Doctor)

Fragmented communication between ICU teams and ODNZ, undermined confidence and donation coordination. Staff advocated for centralised electronic systems to streamline coordination. The presence and visibility of donor coordinators also varied across ICUs, often shaped by historical organisational arrangements. Where coordinators were embedded in daily practice, they provided care continuity, built trust and provided timely guidance. ICUs without their presence relied on link nurses, creating inconsistencies in donation support and confidence. Limited psychological support for families and staff, particularly on weekends of after hours, was also a critical gap, underscoring the need for greater investment in specialist services.

Routine and robust donor identification was another area needing improvement. While some units used structured handover triggers to identify potential donors, others relied on individual discretion, leading to missed opportunities, particularly for tissue donation.

We definitely do miss tissue donation opportunities, because we haven't developed stronger, more robust systems to identify cases. (P4‐Doctor)

Staff also identified gaps in public understanding. The driver's licence system for recording donation wishes was seen as inadequate for supporting informed decision‐making. Staff advocated for a national registry and public awareness campaigns to provide accurate, accessible information that would prompt early, informed conversations in the community.

The tick box when you go and get your license is not really an informed consent process […] we're not sure whether someone really understood the implications of ticking that box in the context of going to renew their license. […] To have a massive public campaign, have a registration set up where people have the information they need, […] discussed it with the people who are important to them, […] that would be really valuable. (P4‐Doctor)

## 6 Discussion

This study explored ICU staff experiences of organ donation discussions, identifying three themes that captured the foundations of an organisational culture that staff appreciate for quality organ donation discussions. The importance of building meaningful relationships with families, managing the emotional demands of donation work and the practical and contextual resources that prepare and sustain staff were examined. The findings offer a strengths‐based perspective of valued practice, systems and behaviours worth sustaining and strengthening.

Staff prioritised early trust‐building through clear, inclusive and emotionally attuned communication before introducing donation. While some literature focuses on consent rates [25, 26], participants advocated for informed and meaningful decision‐making, aligning with broader research that positions trust and connection as central to end‐of‐life conversations [27, 28, 29]. Clinicians who actively listen, attend to explicit and subtle cues and engage in exploratory dialogue foster greater emotional safety and responsiveness [29, 30].

Participants emphasised meeting families where they are culturally, emotionally and clinically, by assessing readiness to discuss donation and calibrating conversation pace and content accordingly. Culture was understood broadly, extending beyond ethnicity to encompass family values, communication styles and priorities. These findings reflect literature showing that cultural sensitivity and emotional responsiveness enhance family engagement with challenging end‐of‐life and organ donation discussions [29, 31, 32]. Adapting communication styles, involving cultural liaisons, supporting prayers and rituals and prioritising building trust are integral to culturally safe and equitable care [10, 33, 34]. Ultimately, ‘good’ donation conversations support decisions that are informed, enduring and feel right to families, regardless of the outcome.

The importance of emotional expression and collegial support in managing the emotional demands of organ donation work was also appreciated among participants. Without support, staff risk compassion fatigue and burnout [35]. Regular peer support, pre‐briefing and debriefing enable staff to reconcile personal and professional values, maintain emotional balance and sustain quality care [36]. Given the emotional labour involved in donor and recipient journeys, informal and structured collegial support is vital for processing emotions and fostering resilience and shared purpose [35, 37, 38, 39, 40]. Participants' openness toward emotional expression and peer support reflects a cultural shift from past clinical norms that often suppressed emotions in the name of professionalism [41]. This normalisation of emotional expression was seen to enhance staff well‐being and the quality of donation practice.

Participants also reflected on the tangible resources within the local context required to prepare staff for effective organ donation discussions. Consistent with previous studies, ongoing, mandated training was viewed as essential for maintaining competence, consistency, confidence and positive attitudes around donation [42, 43, 44, 45, 46]. Seminars, reflective forums and role‐play simulations improve staff confidence and communication skills [42, 44, 46]. Although implementation varied, structured team preparation before approaching families was critical for delivering cohesive and sensitive care. ODNZ guidelines also stress the importance of clear team roles and thorough preparation [47]. Research shows that well‐coordinated communication enables families to feel informed, empowered and emotionally supported during time‐sensitive donation processes [48, 49, 50].

Adequate resource allocation (including protected physician time, on‐call support and workforce expansion) was also essential for sustaining quality organ donation programs that included fostering staff confidence. Insufficient support often leads to rushed care, missed organ donation opportunities, increased burnout and compassion fatigue [6, 16, 51]. Staff frequently invest considerable effort, with higher levels of self‐criticism and unmet expectations can lead to exhaustion and cynicism [52].

Robust donor identification systems were also viewed as a necessity, including accessible donor registries and routine prompts, such as unit handover triggers. Other studies have also highlighted that a strong donor coordinator presence, high levels of staff education and experience in donation practices and a positive ICU culture toward organ donation were facilitators to improve donor identification [16, 53, 54]. Practical tools such as badge buddies, cue cards and screensavers displaying referral criteria have also been recommended to prompt timely identification and support collaborative donation discussions with families [55].

Participants expressed frustration with NZ's current donor indication system, which relies on a simple checkbox at driver's licence registration without educational context, limiting its clinical usefulness. Evidence suggests that pairing easy registration with robust public education that addressed common concerns improves donation discussions and supports positive, informed decision‐making experiences for families [56, 57]. Integrating ODNZ with the national transport agency to create clear, engaging educational tools for drivers could better inform the public, prompt early family discussions and improve awareness of the realities and implications of donation to enhance decision‐making processes in the ICUs.

## 7 Strengths and Limitations

This study drew on the insights of self‐selected, purposively sampled participants with direct experience facilitating organ donation discussions. Multidisciplinary participation and variation in ICU experience supported a nuanced understanding of team dynamics and unit culture. Profession‐specific focus groups helped reduce power imbalances and fostered open, reflective dialogue.

Engagement with rich, diverse data prompted the researcher (L.S.) to shift from a system‐oriented lens to a more interpretive understanding of positive donation culture within ICU settings. Rather than focusing solely on system‐level barriers and enablers, this shift deepened the findings by centring participants' cultural and contextual experiences, highlighting how local values, beliefs and interpersonal dynamics shape organ donation discussions. L.S.'s clinical background supported rapport‐building and contextual understanding, while reflexivity strengthened analytical depth and credibility.

Findings are grounded in tertiary ICU settings with access to donor coordinators or specialised donation link teams, multidisciplinary staff, cultural and religious family support services and established organ donation protocols. These contextual descriptions support assessing transferability to similar ICU environments. However, participants identified with three ethnic backgrounds, with most identifying as NZ European. This limited diversity means findings may not reflect the perspectives and cultural experiences of other groups, whose values and worldviews can shape organ donation practices and family decision‐making. Future research could include rural ICUs, broader ethnic representation and donor coordinators, who play a central role in donation processes.

## 8 Implications

Regular donation‐specific education, multidisciplinary simulation, structured pre‐briefing and debriefing and team‐building exercises could be embedded into routine ICU practice to strengthen staff confidence and family communication. Box 1 outlines practical considerations informed by this study's findings. Organisations should ensure adequate workforce support and robust donor identification systems to facilitate timely, coordinated donation discussions. Nationally, strengthening public education and donor registration pathways may better prepare families for donation decisions before ICU admission.

BOX 1 **Practical considerations to strengthen ICU staff confidence and family communication during organ donation discussions.**

**Table:** 

Recommendation	Practical considerations
Donation‐specific education	Regular education on donor identification, referral processes, communication skills, available resources and roles of ICU and donation staff.
Multidisciplinary simulation	Simulation workshops of organ donation conversations involving ICU doctors, nurses and donation specialists, including challenging family scenarios.
Structured pre‐briefing	Brief meetings before family discussions to clarify clinical information, team roles, communication goals and anticipated family needs.
Structured debriefing	Facilitated reflection after donation discussions to identify learning opportunities, provide emotional support and reinforce staff well‐being.
Team‐building	Joint education, interdisciplinary case reviews and opportunities to strengthen collaboration between ICU and donation teams.

## 9 Conclusion

This study explores the experiences of ICU staff in NZ supporting families through organ donation decisions, highlighting the central role of a positive donation culture in shaping high‐quality practice. Rather than focusing solely on consent, staff prioritised meaningful, inclusive and trust‐based conversations that addressed families' unique needs. Sustaining this approach required collegial support, emotional resilience and access to resources that build confidence and competence. The findings highlight effective current practices and opportunities for targeted support and system‐level improvements.

## Funding

The authors have nothing to report.

## Ethics Statement

Ethical approval was granted by the Auckland Health Research Ethics Committee [Ref: AH26759; Approval date: 18/12/2023], with locality approval obtained from participating hospitals. All participants received written information about the study and were given the opportunity to ask questions prior to participation.

## Consent

Written informed consent was obtained from all participants before conducting focus groups.

## Conflicts of Interest

Rachael Parke is an editor for *Australian Critical Care*.

## Supporting information


**File S1:** Staff focus group topic guide.

## Data Availability

The qualitative data from this study are not publicly available due to confidentiality and ethical considerations assured to participants.
