# Supplementary material for: The Heart of the Matter: Staff Insights Into Organ Donation Conversations
Source: Nurs Crit Care. 2026 Sep 8;31(5):e70668. doi: 10.1111/nicc.70668 (PMC13551057; doi:10.1111/nicc.70668)
Supplement: Supplementary file 1 [file NICC-31-0-s001.pdf]

Supplementary file 1. Staff focus group topic guide

Developed from literature review findings [8]

Introduction of researcher and expression of appreciation to participants

A brief outline of the research purpose and focus group rules

Example statement:

We've gathered today, as a group, to explore your experiences of deceased organ donation conversations with families. The purpose is to understand what ICU staff across various practising levels feel are factors influencing organ donation decisions. We'll be discussing four main areas around knowledge and training, communication, internal factors, and external factors which may help us identify barriers or enablers to facilitating family discussions. It's important we go over the rules for holding a focus group:

- What is said in this focus group, stays in this focus group. Please do not share information shared here with others outside this group. We would like to safeguard each other's privacies.
- There is no right or wrong answer-just the insight that your unique experience brings.
- You do not have to answer all the questions if you do not want to but participation and any opinion you offer will be valued.
- This session will be voice-recorded, you may leave at any time if you wish to.

Opening Karakia | Prayer

Take an opportunity for everyone to introduce themselves and their roles/practising levels in the ICU.

<table>
<tr><th>Literature review-derived questions</th><th>Discussion prompts</th></tr>
<tr><th colspan="2">Theme 1: Important knowledge</th></tr>
<tr><td>1. Can you think of a time when a conversation or approach about organ donation went well?

If so, what do you think made it go well?

2. Can you think of a time when a conversation or approach did not go so well? What do you think made it go not so well?</td><td>● What level of knowledge did you have and how prepared did it help you feel? And Why?
i.e. how well did you understand:
■ brain death testing?
■ what happens for families after they agree to organ donation?
■ The DCDD pathway?

● How accessible or easy is it for you to receive the education and training currently available? And how helpful are they?
● How does your experience in organ donation opportunities help you during donation conversations?
● How do you recognise organ donation opportunities? What helps? And are there any methods in place on your unit to help you?</td></tr>
</table>

<table>
<tr><td></td><td>Internationally, literature findings inform us that training and experience levels influence confidence levels among staff when discussing organ donation with families.
To what extent did your training or experience help with your confidence? What worked well for you or what could have helped?</td></tr>
<tr><td colspan="2">Theme 2: Challenging communication</td></tr>
<tr><td>I want us to focus now, particularly on communication during organ donation discussions.

3. Can you think of a time when communicating with families to discuss organ donation felt challenging? If so, what made it feel difficult?

4. Has anyone had an experience when communicating with families to discuss organ donation felt easy? Would you be able to tell me about that?</td><td>What different roles do you and members of your team usually take when approaching organ donation discussions with families? And why do you think that’s so?
How do you or your team, time these discussions with families? What helps recognise a right time to approach it?
How do you or your team prepare for a conversation about organ donation with families?
How do you deal with the emotions that may arise for you or families during organ donation conversations?
If there ever was a time when organ donation discussions created conflict among the family in your care, how did you manage it?
To what extent does the ethnicity or culture of families influence the way you approach them?
Organ donation often requires multiple teams to communicate with each other too i.e. surgeons, donor coordinators, theatre teams etc.
How do you and your unit navigate communication with these multiple teams while you care for the potential donor patient?</td></tr>
<tr><td colspan="2">Theme 3: Internal determinants</td></tr>
</table>

5. How do you think your personal values, beliefs or emotions impact the organ donation discussions you are involved in?	● What thoughts or emotions surface when you hear the term "organ donation" and why?<br>● Have you ever experienced conflict between your duty as a health care provider and these personal thoughts and emotions or any cultural values you hold? If so, why? And how did you manage that?
**Theme 4: External determinants**	
We've discussed knowledge, communication and our personal values. I want us now to look at the ICU environment we work at.<br><br>This may include the pace of the ICU shift and acuity of the workload, the resources we have access to such as equipment, space, protocols and guidelines or the unit culture/routine.<br><br>6. To what extent does your workload in the ICU unit influence your ability to facilitate and support organ donation discussions with families?	● How do you feel supported to manage it?<br>● In what ways could you be better supported, if any?
7. When caring for the potential donor and facilitating donation decisions, how satisfied are you with the resources you have in your unit?<br>i.e. space, equipment, policies, guidelines etc.? and why?	● What policies or guidelines are you aware of to help you and how helpful/accessible do you find them? And why? (i.e. ODNZ conversation guidelines with families should be available at every ICU)<br>● Given all that we have discussed, how could our current policies/guidelines be improved?<br>● Are there any other types of resources that could benefit the ICUs to facilitate donation discussions with families?
8. How do other staff, or the ICU routine/culture on your unit enable you to approach or facilitate organ donation discussions?	● What sort of routine reminders do you have on the unit to help you recognise organ donation opportunities?<br>● What sort of support can you expect from your team when you are caring for potential donors and facilitating organ donation decisions?

	i.e. are there regular debriefs? Are encouraging comments expressed?<br>● Are there any additional ways your team or the unit routine could better enable you to identify organ donation opportunities and facilitate discussions?<br><br>● Are there any other factors in the ICU environment that affect you when you are planning to approach or facilitate organ donation discussions?
Internationally, many countries have a national organ donation register where healthcare professionals can access the donation wishes of their patients before approaching the family. In New Zealand, we have the driver's license which indicates a donor status, although not all the time.<br><br>9. To what extent does the donation status on the driver's license serve you when caring for the donor family?	● How accessible are the donor statuses of patients' driver's licenses?<br>● How do you usually identify the wishes of patients regarding organ donation?<br>● Do you have any ideas on how to improve identifying the organ donation wishes of our patients?
**Closing questions**<br>● Based on today's discussion, if anything, how else do you feel we could better support ICU staff to approach and facilitate organ donation discussions with families?<br>● Is there anything else anyone would like to say or ask?	
**Conclude focus group by thanking participants for sharing | Closing Karakia | Prayer**	
